# Adapting online learning for Canada's Northern public health workforce

**DOI:** 10.3402/ijch.v72i0.21345

**Published:** 2013-08-05

**Authors:** Marnie Bell, Karen MacDougall

**Affiliations:** 1 Public Health Consultant Public Health Agency of Canada Centre for Public Health Capacity Development Ottawa, Ontario, Canada; 2 Program & Policy Analyst Public Health Agency of Canada Centre for Public Health Capacity Development Ottawa, Ontario, Canada

**Keywords:** e-learning, professional development, continuing education, core competencies for public health, Skills Online, paraprofessional, north or northern, Aboriginal

## Abstract

**Background:**

Canada's North is a diverse, sparsely populated land, where inequalities and public health issues are evident, particularly for Aboriginal people. The Northern public health workforce is a unique mix of professional and paraprofessional workers. Few have formal public health education. From 2009 to 2012, the Public Health Agency of Canada (PHAC) collaborated with a Northern Advisory Group to develop and implement a strategy to strengthen public health capacity in Canada's 3 northern territories. Access to relevant, effective continuing education was identified as a key issue. Challenges include diverse educational and cultural backgrounds of public health workers, geographical isolation and variable technological infrastructure across the north.

**Methods:**

PHAC's Skills Online program offers Internet-based continuing education modules for public health professionals. In partnership with the Northern Advisory Group, PHAC conducted 3 pilots between 2008 and 2012 to assess the appropriateness of the Skills Online program for Northern/Aboriginal public health workers. Module content and delivery modalities were adapted for the pilots. Adaptations included adding Inuit and Northern public health examples and using video and teleconference discussions to augment the online self-study component.

**Results:**

Findings from the pilots were informative and similar to those from previous Skills Online pilots with learners in developing countries. Online learning is effective in bridging the geographical barriers in remote locations. Incorporating content on Northern and Aboriginal health issues facilitates engagement in learning. Employer support facilitates the recruitment and retention of learners in an online program. Facilitator assets included experience as a public health professional from the north, and flexibility to use modified approaches to support and measure knowledge acquisition and application, especially for First Nations, Inuit and Metis learners.

**Conclusions:**

Results demonstrate that appropriate adaptations to online professional development can provide practical, accessible means for a wide range of Northern/Aboriginal public health workers to acquire core competencies for public health.

While public health human resource capacity is a challenge nationwide, it is particularly acute in Canada's 3 northern territories: Yukon, Northwest Territories and Nunavut. There are not enough skilled public health practitioners (professionals and paraprofessionals) or continuing education opportunities for existing staff who work in population health assessment, health protection, health/wellness promotion, disease and injury prevention, surveillance and emergency preparedness.

In his landmark report following the 2003 SARS crisis, Naylor (1) recommended the strengthening of Canada's public health workforce. In their 2005 Pan-Canadian Framework (2), the Joint Task Group on Public Health Human Resources recommended core competencies for interdisciplinary public health practice as a foundational building block to strengthen and develop the public health workforce.

In 2007, following an extensive nationwide consultation, the Public Health Agency of Canada (PHAC) launched the Core Competencies for Public Health in Canada: Release 1.0 (3). Thirty six competency statements organized under 7 categories: (a) public health sciences, (b) assessment and analysis, (c) policy and program planning, implementation and evaluation, (d) partnerships, collaboration and advocacy, (e) diversity and inclusiveness, (f) communication and (g) leadership, define the essential knowledge, skills and attitudes required for public health practice.

PHAC's Skills Online program provides competency-based continuing education for public health professionals through a series of facilitated and self-directed Internet-based modules (4). Currently Skills Online offers learners asynchronous access to 10 facilitated modules 3 times per year, and continuous access to 2 self-directed modules. Module content aligns with the core competencies. Facilitators, experienced in public health, guide learners through module material, answer content-related questions, encourage online discussion and provide feedback on learning exercises. From 2002 to 2011, over 6,500 learners from across Canada completed over 15,000 Skills Online modules (5). Regularly scheduled module reviews assure that module content remains current and relevant. New module development is based on clearly identified learning needs. On-going learner feedback and scheduled case studies contribute to continuous program improvement. Skills Online is a Canadian leader in online public health continuing education and in 2011 received the silver award for training excellence in the e-learning category from the Canadian Society for Training and Development.

Could Skills Online contribute to strengthening the competencies of northern public health practitioners? What adaptations might be needed for module delivery to paraprofessionals? Is there a need for a new innovation in skill development to better match the diversity within the public health workforce across the North? These are questions that stimulated PHAC to pilot-specific modules with groups of Northern learners.

## Pilot studies

### Background

The Canadian North is a vast, isolated, culturally diverse, sparsely populated land, where inequalities and public health issues are evident, particularly for Aboriginal peoples – the First Nations, Inuit and Metis. Aboriginal people constitute significant proportions of the northern population: 25% in Yukon, 50% in Northwest Territories and 85% (primarily Inuit) in Nunavut (6). The public health workforce in the North is a diverse mix of practitioners working together at community, regional and territorial levels. Primary employers are the territorial governments. However, non-governmental organizations also hire staff and partner in public health service delivery. The most diversity is seen in small communities, where professionals, primarily registered nurses, and paraprofessionals with varied educational backgrounds and skills, as well as diverse ethnic and language backgrounds, find themselves working together in a model of service delivery that integrates primary care and public health.

Paraprofessional public health workers have many job titles, but similar roles and responsibilities, and they share common characteristics. Their primary roles are health (or wellness) promotion, disease and injury prevention, and community capacity building through various population health initiatives such as tobacco cessation, safer sex and diabetes awareness. Paraprofessionals are usually members of the communities they serve and share ethnicity, language, socio-economic status and life experiences with other community members. They are often selected by the community and by virtue hold a position of respect and trust within the community. Preparation for paraprofessional public health practice varies across the North. While some territories have training programs at the post-secondary level, not all paraprofessionals are trained to the same level.

Health professionals are primarily recruited from southern jurisdictions despite a concerted effort to increase the number of northerners, especially Aboriginal people, in health careers. Due to the broad scope of employment, professionals, especially nurses, are recruited primarily for their emergency and acute care skills rather than competence in public health. Recruitment and retention of health and public health professionals in the North is linked to many quality of life/work life factors. High turnover rates, the repeated loss of institutional memory and the necessity to constantly backfill positions are problematic (7–9).

From 2009 to 2012, the PHAC collaborated with a Northern Advisory Group to develop and implement a strategy to strengthen public health capacity in Canada's 3 northern territories. Access to relevant, effective continuing education was identified as a key issue. Challenges include diverse educational and cultural backgrounds of public health workers, geographical isolation and variable technological infrastructure across the North.

A strategic effort ensued to increase access to PHAC's Skills Online *e learning* modules and to address the challenges through a series of 3 pilot interventions. This article focuses on the evaluations of 3 pilots.

### Methods

PHAC's Skills Online program offers Internet-based continuing education modules for public health professionals. In partnership with the Northern Advisory Group, PHAC conducted 3 pilots between 2008 and 2012 to assess the appropriateness of the Skills Online program for Northern/Aboriginal public health workers, including paraprofessionals. Module content and delivery modalities were adapted for the pilots. Adaptations included adding Inuit and Northern public health examples to online content and using video and teleconference discussions to augment the online self-study component.

These Skills Online pilots were conducted in fall 2008, winter 2010 and winter 2012. Common design elements included:

- Targeted solicitation of Northern learners and in particular, paraprofessionals with Aboriginal ethnicity from the public health workforce who would not necessarily self-register for Skills Online modules.
- Assignment of a trained facilitator with Northern and Aboriginal public health experience.
- Adaptation of module content and/or delivery modality to better meet learning preferences and specific knowledge gaps of the learner group.

Table I summarizes the adaptations in design and delivery.

**Table I:** Target groups and design/delivery adaptations for Northern Skills Online pilots, 2008, 2010 and 2012

Pilot module	Timeframe	Target group	Adaptations
Basic Epidemiological Concepts fundamentals of epidemiology application of basic epidemiological principles (Core Competencies for Public Health domains 1.0 Public Health Sciences and 2.0 Assessment and Analysis)	Fall 2008	Inuit paraprofessionals & First Nations professionals (n = 5)	no content changes selected facilitator weekly teleconferences organizational support
Basic Epidemiological Concepts	Winter 2010	Northern paraprofessionals & professionals (focus: First Nations, Inuit & Metis) (n = 14)	no content changes selected facilitator enhanced facilitator support (teleconferences and personal contact)
Introduction to Public Health in Canada purposes, functions and approaches of public health who is involved how the system operates history of public health in Canada (Core Competencies for Public Health domains 1.0 Public Health Sciences)	Winter 2012	Inuit paraprofessionals (n = 13)	content adaptations selected facilitator (professional + paraprofessional) weekly teleconferences employer support

Learnings from previous pilots informed the design of subsequent pilots. The evolving series of pilots with slightly different participant profiles prevented meaningful comparative measurement of the impacts and outcomes of the interventions. Small sample size limited generalization.

The pilot evaluations used qualitative and quantitative measures to assess relevancy, effectiveness and design and delivery of the pilots. Data sources included focus groups, online module feedback surveys, facilitators’ journal log and notes, Pilot Planning Group feedback and one-on-one telephone interviews.

This article summarizes the key findings from the pilots and does not present extensive individual pilot data except to illustrate significant learnings.

### Findings

#### Learner solicitation

Targeted solicitation proved to be a successful way to recruit learners for the pilot modules. Additionally, oversubscription was permitted to offset the higher than average attrition rates for Northern learners and maintain a desirable class size. The average Skills Online attrition rate for English learners is 30% (7), whereas the attrition in the second northern pilot was 60 and 77% in the third pilot.

#### Learner retention

The 2 most prevalent challenges to completing a Skills Online module were a lack of time due to workload and lack of time due to personal time conflicts, which were also identified in prior attrition study findings (10). The third pilot additionally identified technological challenges such as broadband infrastructure and computer literacy as barriers to participation and module completion. While the infrastructure issue was systemic, the computer literacy issues were at the individual level and amenable to mentoring and onsite support.

#### Facilitation

In all 3 pilots, learners consistently rated the attributes of the selected facilitators as enablers to their learning. Facilitators were selected based on their knowledge and prior experience with Northern practice and Aboriginal public health issues. Learners appreciated the level of expertise and ability of facilitators to connect to their reality.

Facilitators also need to be flexible and innovative to address the challenges that diversity within learning groups presented to e-learning.

#### Diversity

Diversity within learning groups was both a benefit and a challenge, especially for facilitators. In the second pilot, the learners’ educational backgrounds were diverse especially between professionals and paraprofessionals. While there were pedagogical benefits that resulted when the learners actively engaged and shared knowledge with others, the facilitator was challenged to find time to provide the additional individual support required for paraprofessionals to grasp a new concept. The following comment from the facilitator of the second pilot highlights the need for multi iterations of a new concept among paraprofessionals.

How to evaluate assignments? I'm finding a wide variation in the writing styles among the participants, (specifically those without a college/university degree). For these individuals I often provide feedback and then the participant will provide some type of response. Most often, it takes on a series of questions and answers. I then attempt to evaluate whether the participant has understood the concept. (11)

This finding spawned a recommendation to assign an additional facilitator for paraprofessional or mixed paraprofessional/professional learning groups to assist with coaching when warranted. A co-facilitation model was tested in the third pilot, composed solely of paraprofessionals from Nunavut. The paraprofessional learners in the pilots generally had lower health literacy levels than the professional learners. This required altering learning assessment methods to include oral in addition to written modes, using multi-methods to convey messages and proactively looking for signs in a virtual environment that might indicate disengagement. Flexibility of facilitators in tailoring and adapting communication, engagement and participatory strategies to fit with learning styles and literacy levels increased retention and module completion.

Computer literacy levels also varied within pilots, but were especially noticeable in the third pilot. Some learners lacked confidence in using the computer, which appeared to be associated with lower educational levels and older age. This lack of prerequisite skill prevented them from even attempting the online portion of the module, yet they did call in for weekly teleconferences. This required the facilitator to summarize the lesson for those who did not access content prior to the teleconference.

#### Technological issues

Technological challenges surfaced in all pilots, but most significantly in the third pilot. In this pilot, all participants lived in Nunavut, where communities are solely reliant on satellite-based Internet signal. Slow downloading speed and connectivity issues challenged learner participation in the pilot. Additionally, hardware and software issues unidentified prior to the pilot were barriers for learners that had to be addressed during the pilot. For example, computers were upgraded and programs were installed to enable access to the e-learning platform.

Overall system infrastructure varies across the 3 territories. Generally, technological infrastructure stability and speed increases as you travel west across Canada's North which makes online learning easier for those living in Yukon and in larger NWT communities, where Internet infrastructure is generally better. Some contributors to this evaluation felt that the Internet connections available to them were inadequate to enable high-speed Internet browsing and downloading. Until this issue is addressed, considerations to circumvent the need to download files are critical to prevent withdrawal from online courses.

An interesting phenomenon in the third pilot was that 85% of the learners participated in the pilot in some way, either by facilitated teleconference discussions (synchronous interactive) or by doing the facilitated online study (asynchronous self-learning) or by participating in both synchronous and asynchronous activities. Despite technological challenges related to broadband infrastructure and personal computer literacy, the pilot group learners remained eager to participate in the module using the technology they were most familiar with – the teleconference.

#### Content adaptations

Original Skills Online module content, which already included 1 Northern epidemiological example, was used for the first 2 pilots. Facilitators attempted to introduce more Aboriginal and Northern examples through discussion forums and teleconferences but this was not readily recognized in the evaluation ratings as a useful adaptation. Learners consistently stated that the modules could be improved with the addition of relevant Aboriginal and Northern examples to illustrate concepts presented in the online module content. This finding was consistent with Skills Online pilots conducted with Caribbean learners who desired more tropical public health examples (12).

The third pilot incorporated a customized version of a module designed to address a specific learning gap of the target audience. The content was adapted with the substitution of Inuit and Northern scenarios and examples, as well as the addition of Inuit-specific web-based exploratory activities which learners rated highly for relevance. One learner noted the relevance of the content adaptations to daily work:

I was glad to know that we have a website for Inuit health statistics. This was very useful. I forwarded that website to a teacher so students could look up statistics for their region. I also told my co-workers about the website on Inuit health statistics and they were very interested. (13)

#### Delivery adaptations

The Skills Online pilots demonstrated that Aboriginal paraprofessional learners require more individualized support and that telephone conversations/conferences are often more effective than electronic communication mechanisms in establishing whether messages and concepts are received and understood.

The first 2 pilots relied on enhanced facilitation to provide adequate support for diverse learners within mixed learner groups. Given the diversity of the learners and learning preferences in the pilots, facilitators needed to be flexible, innovative and able to use a variety of engagement and assessment techniques. In the third pilot, module delivery was significantly adapted by adding facilitation to what was originally intended to be a strictly online self-study. The delivery was adapted by using 2 facilitators – a content expert plus a context expert, adding an initial ice breaker videoconference, and spacing the online self-study module over a 4-week period with the opportunity for weekly interactive telephone discussions.

The discussion questions were embedded in the online content to enable learners to prepare for the synchronous discussion and sharing of their stories. As story telling is a traditional knowledge exchange modality for Aboriginal people, this strategy proved successful in bolstering understanding of concepts, in increasing self-esteem and confidence in their role so that they feel comfortable explaining their role to others with reinforced self-worth. One pilot participant stated:

Sometimes I feel I haven't accomplished anything but when I have to answer questions, I realize that I have done something. It makes me feel good when I review what I do in my job. I had lots to share with others. (13)

#### Employer support

In all pilots, employer support was cited as an enabling factor associated with learner retention and successful module completion. The support of employers for staff professional development can be demonstrated in a variety of ways such as reimbursing course costs, allowing study to be done at work, providing lieu time when study is done outside of work time, allowing use of work computers, providing technical support, mentoring and bestowing recognition. In some cases, while employer's intentions were good, workload interfered with being able to do online study during work time. Learners acknowledged that employers who were flexible and supportive of finding solutions to barriers were instrumental to learner retention in the module.

#### Participation trends

**Fig. 1:**
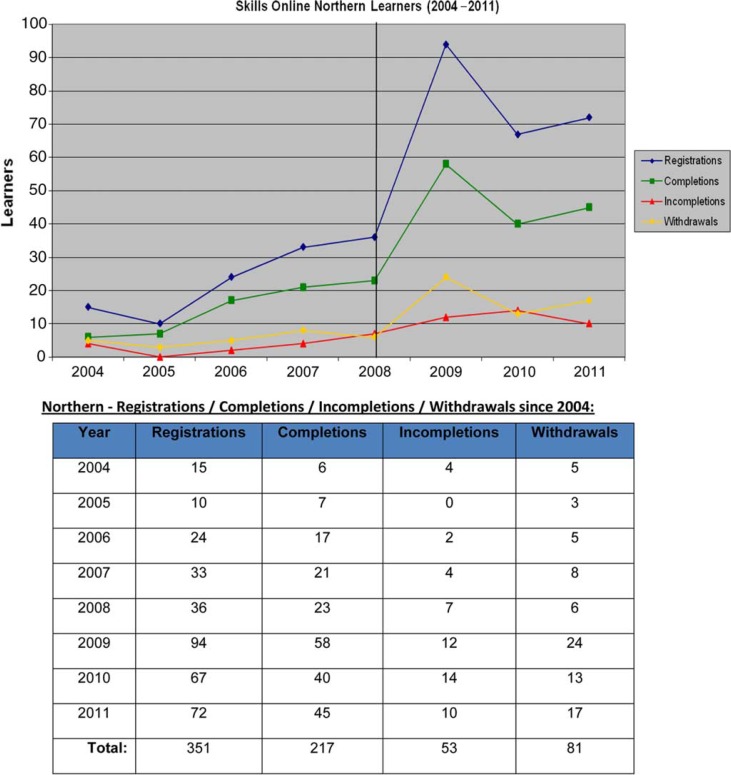
Number of Skills Online learners from Canada's North (2004–2011). Source: Skills Online registration database.

Since 2009, as part of a workforce development project, PHAC has supported a focused effort to promote participation in the Skills Online program in Canada's North. Public health professionals were recruited both through on-going mainstream promotion and through targeted solicitation for the pilots. Paraprofessionals, who normally would not enrol in Skills Online, were recruited for the pilots only. All efforts contributed to a general heightened Skills Online awareness and enrolment in the North. The number of registrations and completions tripled in the first year of the northern workforce development project when compared to the 2008 baseline. In subsequent years, the registration numbers stabilized at twice that of baseline. Figure 1 shows the trend in the number of Northerners who registered, completed, withdrew or did not complete modules over a 7-year period.

In general, the attrition levels for all Northern learners remain a concern at 40% (5), 10% higher than the average attrition rate for Canadian learners (5). Learning from the pilots provides insight into measures that can be taken to increase retention and completion of online modules in a northern environment.

## Lessons learned

Three key lessons learned were:

- Know your learners. Adapt content and/or delivery modality accordingly. Northern public health practitioners, both professional and paraprofessional, represent a diversity of educational backgrounds, literacy levels, learning preferences, computer skills and practice environments. Module/course content and/or delivery modalities may need to be adapted to maximize retention and completion.
- Know your learner's environment. Be proactive and identify technological challenges and find innovative solutions before online course start dates. Inequities may exist in the availability and quality of technology to enable e-learning. If you know this in advance, you can address the issues (e.g. hardware and software upgrades) prior to courses starting and make adaptations to overcome systemic barriers (e.g. saving videos on USB sticks to view on personal computers rather than downloading from the Internet).
- Employer support enables completion of e-learning. Ensure employers are aware of this factor. Learners appreciate work time and use of office equipment to do online study. Lieu time and use of laptop computers, if study is done partly or wholly outside of work, is also effective. Encouragement and recognition from supervisors who show genuine interest in what employees are learning contributes to the retention of learners and application of this learning to their work.

## Conclusions

Overall system infrastructure varies across the 3 territories. Generally, technological infrastructure stability and speed increases as you travel west across Canada's North which makes online learning easier for those living in Yukon and in larger NWT communities, where Internet infrastructure is generally better. Some contributors to this evaluation felt that the Internet connections available to them were inadequate to enable high-speed Internet browsing and downloading. Until this issue is addressed, considerations to circumvent the need to download files are critical to prevent withdrawal from online courses.

While not a perfect fit for all Northern learners, Skills Online can be adapted for a more successful online learning experience. Participation can be augmented with support customized to meet the needs of diverse learning groups. Facilitated learning groups are more appropriate for paraprofessional groups than independent self-study. Advantages exist for learners when knowledgeable, culturally sensitive and committed public health experts facilitate online learning. In some Skills Online pilot cases, individuals did better when they had a mentor on site to help them navigate the module and become more confident in using a computer. Learning is enhanced when content is customized and there are provisions made for interactive synchronous activities that employ visual as well as audio tools. Finally, it is worth noting that computer proficiency, the availability of computers, software and technological support, graduation from high school and previous online learning experience were associated with Skills Online success in the 3 pilots.

Participation trends in Skills Online (Fig. 1) illustrate a persistent issue with attrition amongst Northern learners. The pilots afforded some guidance on adaptations to e-learning that can increase the retention of Northern learners but further study is warranted.

In Canada's North, a blended approach that enhances the more traditional pedagogy with online delivery has the best potential to not only support public health practitioners to increase their core competencies, but also to increase computer literacy. As the territorial colleges move to incorporate more e-learning into their course delivery modalities, these findings are informative. The findings may also be useful to other circumpolar countries with similar geography, workforce demographics and technological challenges.
